# Treatment of septic arthritis and acute osteomyelitis

**DOI:** 10.1186/1546-0096-12-S1-I30

**Published:** 2014-09-17

**Authors:** Heikki Peltola

**Affiliations:** 1 University of Helsinki; 2 Infectious Diseases, Helsinki, Finland

## 

Acute hematogenous bone and joint infections, septic arthritis, and osteomyelitis with or without adjacent septic arthritis, are rare among children in a standard Western setting, but still potentially devastating diseases, as even deaths have been reported recently. Foir this reason, and in part due to historical reasons, the treatment has comprised of months-long courses of antibiotics, started intravenously for at least a week, and aggressive surgery. Recent prospective and randomized trials have shown that a 2-4-day parenteral course, completed orally to a total duration of 10-14 days for septic arthritis and of 3 weeks for osteomyelitis, heals the great majority of cases, provided large-enough doses of a well-absorbing antibiotic, and a four-times-daily (*qid*) regimen is used. *Staphylococcus aureus -* the most common causative agent in osteoarticular infections - is the primary target for treatment. For methicillin-susceptible strains, first-generation cephalosporins, clindamycin, and staphylococcal penicillins are first-line antibiotics of which clindamycin has retained activity even for most cases due to methicillin-resistant *S.aureus.* This said, instead of clindamycin, beta-lactam antibiotics are effective also against *Kingella kingae.* The role of surgery in uncomplicated cases is minor, even in cases of shoulder or hirp arthritis, as most children recover uneventfully with no greater intervention than diagnostic bone or joint aspiration. Routine arthrotomy seems unnecessary even in hip or shoulder arthritis. However, each patient needs an individual approach, and a deviation from the general treatment lines should be executed when the conditions so dictate.

## Disclosure of interest

None declared.

